# Linezolid pharmacokinetics in MDR-TB: a systematic review, meta-analysis and Monte Carlo simulation

**DOI:** 10.1093/jac/dky096

**Published:** 2018-03-23

**Authors:** James Millard, Henry Pertinez, Laura Bonnett, Eva Maria Hodel, Véronique Dartois, John L Johnson, Maxine Caws, Simon Tiberi, Mathieu Bolhuis, Jan-Willem C Alffenaar, Geraint Davies, Derek J Sloan

**Affiliations:** 1 Wellcome Trust Liverpool Glasgow Centre for Global Health Research, Liverpool, UK; 2 Institute of Infection and Global Health, University of Liverpool, Liverpool, UK; 3 Africa Health Research Institute, Durban, South Africa; 4 Department of Molecular and Clinical Pharmacology, University of Liverpool, Liverpool, UK; 5 Institute of Translational Medicine, University of Liverpool, Liverpool, UK; 6 Liverpool School of Tropical Medicine, Liverpool, UK; 7 Public Health Research Institute, New Jersey Medical School, Rutgers, The State University of New Jersey, Newark, NJ, USA; 8 Department of Medicine, Case Western Reserve University School of Medicine, Cleveland, OH, USA; 9 University Hospitals Case Medical Center, Cleveland, OH, USA; 10 Birat-Nepal Medical Trust, Lazimpat, Kathmandu, Nepal; 11 Department of Infection, Barts Health National Health Service Trust, London, UK; 12 Department of Clinical Pharmacy and Pharmacology, University of Groningen, Groningen, The Netherlands; 13 School of Medicine, University of St Andrews, St Andrews, UK

## Abstract

**Objectives:**

The oxazolidinone linezolid is an effective component of drug-resistant TB treatment, but its use is limited by toxicity and the optimum dose is uncertain. Current strategies are not informed by clinical pharmacokinetic (PK)/pharmacodynamic (PD) data; we aimed to address this gap.

**Methods:**

We defined linezolid PK/PD targets for efficacy (*f*AUC_0–24_:MIC >119 mg/L/h) and safety (*fC*_min_ <1.38 mg/L). We extracted individual-level linezolid PK data from existing studies on TB patients and performed meta-analysis, producing summary estimates of *f*AUC_0–24_ and *fC*_min_ for published doses. Combining these with a published MIC distribution, we performed Monte Carlo simulations of target attainment.

**Results:**

The efficacy target was attained in all simulated individuals at 300 mg q12h and 600 mg q12h, but only 20.7% missed the safety target at 300 mg q12h versus 98.5% at 600 mg q12h. Although suggesting 300 mg q12h should be used preferentially, these data were reliant on a single centre. Efficacy and safety targets were missed by 41.0% and 24.2%, respectively, at 300 mg q24h and by 44.6% and 27.5%, respectively, at 600 mg q24h. However, the confounding effect of between-study heterogeneity on target attainment for q24h regimens was considerable.

**Conclusions:**

Linezolid dosing at 300 mg q12h may retain the efficacy of the 600 mg q12h licensed dosing with improved safety. Data to evaluate commonly used 300 mg q24h and 600 mg q24h doses are limited. Comprehensive, prospectively obtained PK/PD data for linezolid doses in drug-resistant TB treatment are required.

## Introduction

TB remains a major global health problem, with ∼10.4 million cases and 1.7 million deaths in 2016.^1^ Although worldwide incidence and mortality have slowly declined over the last 30 years, the emergence of antibiotic-resistant TB threatens further progress. MDR-TB, defined as resistance to both rifampicin and isoniazid, and rifampicin-resistant (RR) TB (often diagnosed in settings where genotypic and or/phenotypic drug susceptibility testing to isoniazid is not available) are more challenging to manage. There were 600 000 estimated cases of RR-TB or MDR-TB worldwide in 2016, with success rates (cure and treatment completion) of ∼50%.^1^ Outcomes are particularly poor for MDR-TB patients with additional resistance to key second-line drugs (any fluoroquinolone and at least one second-line injectable agent), classified as XDR-TB.^1–4^

Treatment of RR-TB or MDR-TB requires prolonged administration of multidrug regimens including second-line antibiotics with reduced efficacy and higher toxicity than first-line drugs.^5^^,^^6^ High rates of clinical failure, compounded by a rising incidence of second-line drug resistance and regular treatment-limiting toxicities, have prompted increased use of the oxazolidinone linezolid to design adequate regimens. Although currently licensed for use in Gram-positive bacterial infections, linezolid has bactericidal activity against *Mycobacterium tuberculosis* and has been repurposed as a class C, core MDR-TB drug.^5–8^ The standard dose for treatment of Gram-positive infections in adults is 600 mg twice daily (q12h) for a maximum of 28 days, but the duration required for MDR-TB or RR-TB treatment is much longer. Whilst addition of linezolid to RR-TB or MDR-TB treatment can improve outcomes, prolonged administration is often limited by toxicity.^9–11^ Myelosuppression (particularly thrombocytopenia) is common. Peripheral and optic neuropathy, hepatotoxicity, lactic acidosis and hypoglycaemia are rarer adverse effects, but can be serious (and in the case of neuropathies, irreversible) when they occur.^12^^,^^13^ Toxicity from linezolid in TB treatment regularly necessitates dose reduction, but the optimal safe, efficacious dose remains unknown.

In healthy volunteers, the plasma pharmacokinetics (PK) of linezolid are 31% protein binding, excellent tissue penetration, plasma *C*_max_ of 15–27 mg/L, *T*_max_ of 0.5–2 h and a half-life of 3.4–7.4 h.^14^ However, the PK profile varies between patient populations; for instance, critically ill patients have increased levels of free linezolid associated with hypoalbuminaemia, reduced renal clearance with low body weight and markedly increased inter-patient variability.^15–17^ The PK profile of linezolid in TB patients is poorly characterized and dosing has never been informed by an analysis of how successfully different doses might attain target PK/pharmacodynamic (PD) parameters for efficacy and safety.

We defined PK/PD efficacy and safety targets for linezolid in clinical TB treatment from the literature and conducted a meta-analysis of published data collected during therapy to generate summary estimates of key secondary PK parameters: *f*AUC_0–24_ and *fC*_min_. Finally, we simulated attainment of the PK/PD targets on the basis of the summary estimates obtained and a published MIC distribution.

## Methods

### Identifying PK/PD targets

There are no universally accepted PK/PD targets to maximize efficacy and safety of linezolid in TB therapy. In general, the AUC_0–24_:MIC ratio is the PK/PD parameter most predictive of the activity of anti-tuberculous drugs.^18^ For linezolid, some hollow-fibre infection model (HFIM) and *ex vivo* blood culture data suggest that *T*_>MIC_ may influence efficacy against *M. tuberculosis*, but more extensive *in vitro*, murine and human early bactericidal activity (EBA) studies support AUC_0–24_:MIC as the main parameter of interest.^19–22^ HFIMs corroborate clinical data from Gram-positive infections, which suggest an efficacy target of *f*AUC_0–24_:MIC >100–119 mg/L/h. We used the more conservative threshold of 119 mg/L/h as the efficacy target for our simulations.^20^^,^^23–26^

Linezolid clinical toxicity studies are mainly limited to <28 days. Given the cumulative nature of linezolid toxicity, these cannot inform PK/PD targets during prolonged therapy. Amongst the PK parameters, most evidence exists for a relationship between *C*_min_ and toxicity.^15^^,^^27^ In the only clinical study conducted in the context of prolonged TB therapy, all patients with *C*_min_ >2 mg/L developed an adverse event (principally thrombocytopenia) versus less than half of those with *C*_min_ <2 mg/L.^28^ We used *fC*_min_ <1.38 mg/L (equivalent to a total *C*_min_ of 2 mg/L) as the safety target for our simulations.

### Systematic review and meta-analysis of linezolid PK data during TB therapy

To produce summary estimates for *f*AUC_0–24_, and *fC*_min_ for all dosage regimens currently described, we extracted data from all randomized controlled trials or observational studies published in the English language on adult (>16 years) TB patients (any resistance pattern) to whom linezolid was administered for at least 3 days and serum concentrations (at least *C*_max_ and *C*_min_ or AUC_0–24_) were assessed using HPLC and reported disaggregated by dose. Single-study data for more than one dosage (mg) in the same patient was permitted, so long as a minimum washout period of 1 week had taken place. To ensure focus on dosages for which a basic minimum of PK evidence was available, we excluded dosages for which <10 total patients, across studies, were identified.

We searched MEDLINE (1990 to December 2017), EMBASE (1990 to December 2017), The International Union Against Tuberculosis and Lung Disease conference abstracts and American Thoracic Society conference abstracts, using the search terms: Tuberculosis AND (Linezolid OR Oxazolidinone* OR PNU-100766 OR U-100766). This search was supplemented by hand searching the reference lists of identified studies and selected reviews. Authors were contacted to clarify missing or inconsistent data and, if needed, for individual-level PK data.

We constructed time–concentration curves to calculate *f*AUC_0–24_ using the trapezoid rule.^29^*f*AUC_0–24_ and *fC*_min_ data were normally distributed, hence the meta-analysis and Monte Carlo simulations used means and standard deviations as summary descriptors for all studies. If PK results were not otherwise available, data were extracted from published graphs using digitizing software (Plot Digitizer, version 2.5.0). Meta-analysis was conducted using the metafor package in R for Windows, version 3.2.2, to provide a summary mean *f*AUC_0–24_ and *fC*_min_, 95% CI and *I*^2^ statistic for heterogeneity. To emphasize the importance of the heterogeneity of the data, we allowed meta-analysis at any level of heterogeneity.

### Monte Carlo simulation

Using the summary PK estimates identified, we modelled PK/PD target attainment from 100 000 simulated patients at each dose for which data were available. WT linezolid MIC distributions were derived from previously published data in drug-susceptible TB. Briefly, this distribution describes the linezolid MIC results from the isolates of 78 consecutive TB patients in Sweden who had no resistance to any first-line or major second-line drugs. The linezolid MICs ranged from 0.125 to 0.5 mg/L (comprising 1 isolate with an MIC of 0.125 mg/L, 61 isolates with an MIC of 0.25 mg/L and 16 isolates with an MIC of 0.5 mg/L).^30^ There are no published linezolid MIC distributions in RR-TB or MDR-TB. However, MIC values covering 50% and 90% of isolates (MIC_50_ and MIC_90_) in MDR-TB have been reported as 0.25–0.5 and 0.25–1 mg/L, respectively, which is consistent with the WT distribution we used.^31–33^ We assumed a log normal distribution for *f*AUC_0–24_, *fC*_min_ and *f*AUC_0–24_:MIC. We simulated *fC*_min,_*f*AUC_0–24_ and MIC for 100 000 virtual patients in R for Windows. The pnormGC function in the tigerstats package was used to calculate and produce plots of the attainment of the PK/PD targets. We treated the *f*AUC_0–24_ and MIC variables as independent of one another. For doses with high levels of heterogeneity (*I*^2^ >50%) we performed a sensitivity analysis, imputing each study at these doses into the simulation independently to assess the impact of this heterogeneity on target attainment.

## Results

### Meta-analysis of existing linezolid PK data in TB therapy

**Figure 1.**
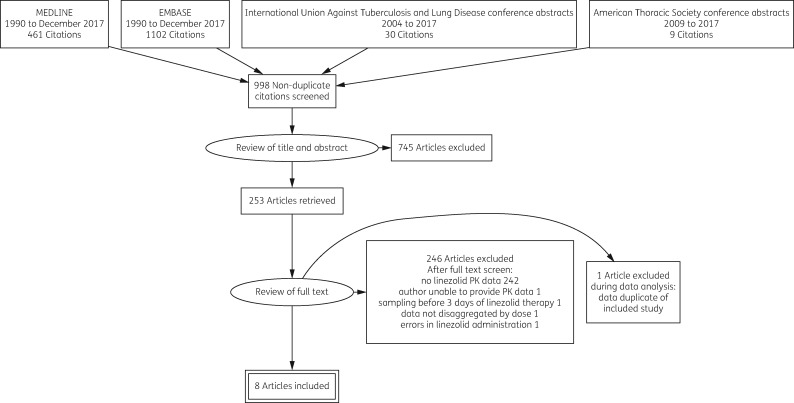
PRISMA flowchart of included and excluded studies for the meta-analysis of existing linezolid PK data in TB therapy.

**Figure 2.**
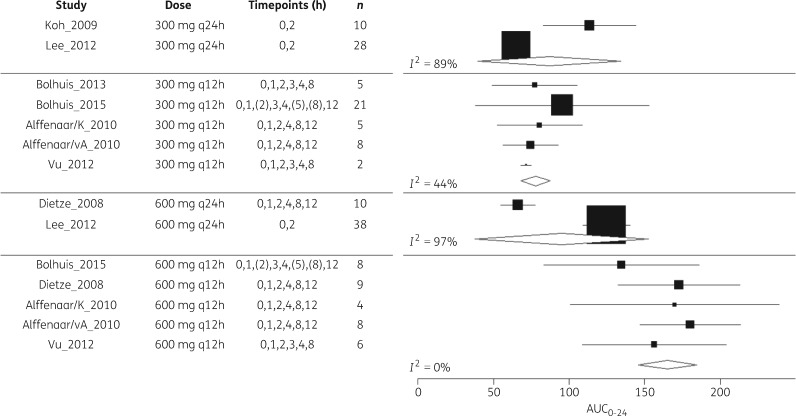
Forest plot of included studies for meta-analysis of *f*AUC_0–24_ at different doses of linezolid. Sampling timepoints in brackets were not assessed for all patients.

**Figure 3.**
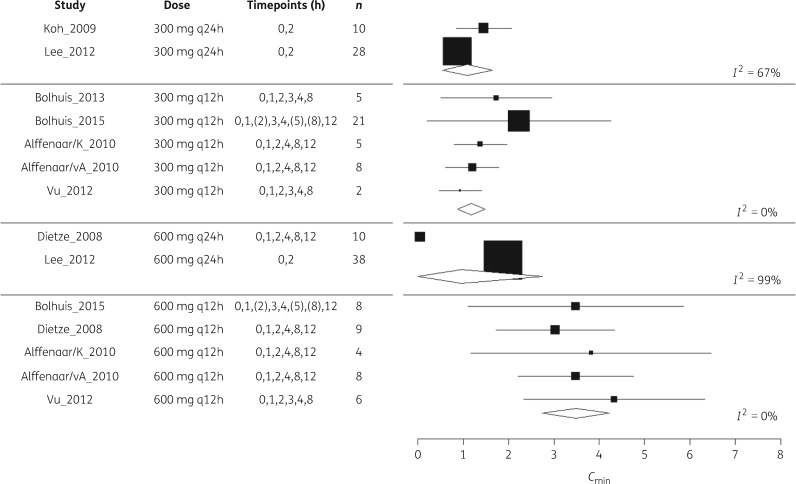
Forest plot of included studies for meta-analysis of *fC*_min_ at different doses of linezolid. Sampling timepoints in brackets were not assessed for all patients.

We screened 1602 citations and eight studies were suitable for meta-analysis. Reasons for inclusion and exclusion are provided in the PRISMA diagram (Figure 1). Included studies are summarized and disaggregated by dose in Table 1. We obtained individual participant-level data for all of these studies. Data were combined using a random effects model; forest plots are provided in Figures 2 and 3. Summary *f*AUC_0–24_ and *fC*_min_ means and standard deviations are provided for each dose in Table 1.

**Table 1.** Meta-analysis of *f*AUC_0–24_ (mg/L) and *fC*_min_ (mg/L) for different doses of linezolid in TB therapy

	Sampling timepoints (h)	Number of participants sampled	*f*AUC_0–24_ mean	*f*AUC_0–24_ SD	*fC*_min_ mean	*fC*_min_ SD
300 mg q24h						
Koh and Shim, 2009^34^	0, 2	10	113.56#	49.33#	1.45ǂ	0.98ǂ
Lee *et al.*, 2012^11^	0, 2	28	64.91*	22.59*	0.87*	0.61*
summary			86.92	149.27	1.09	1.73
300 mg q12h						
Bolhuis *et al.*, 2015^35^	0, 1, (2), 3, 4, (5), (8), 12	21	95.45*	41.60*	2.23*	1.47*
Bolhuis *et al.*, 2013^36^	0, 1, 2, 3, 4, 8	5	77.27*	32.05*	1.73*	1.40*
Alffenaar *et al.*, 2010^37^	0, 1, 2, 4, 8, 12	5	80.51*	32.22*	1.37*	0.66*
Alffenaar *et al.*, 2010^38^	0, 1, 2, 4, 8, 12	8	74.53*	26.54*	1.20*	0.85*
Vu *et al.*, 2012^39^	0, 1, 2, 3, 4, 8	2	71.58*	2.49*	0.93*	0.34*
summary			77.82	31.46	1.18	0.94
600 mg q24h						
Dietze *et al.*, 2008^21^	0, 1, 2, 4, 8, 12	10	66.10*	18.24*	0.05*	0.14*
Lee *et al.*, 2012^11^	0, 2	38	124.75*	48.74*	1.88*	1.19*
summary			95.18	203.16	0.96	6.34
600 mg q12h						
Bolhuis *et al.*, 2015^35^	0, 1, (2), 3, 4, (5), (8), 12	8	134.67	64.17	3.48	2.97
Dietze *et al.*, 2008^21^	0, 1, 2, 4, 8, 12	9	172.75*	61.99*	3.03*	2.00*
Alffenaar *et al.*, 2010^37^	0, 1, 2, 4, 8, 12	4	169.87*	70.53*	3.82*	2.71*
Alffenaar *et al.*, 2010^38^	0, 1, 2, 4, 8, 12	8	180.13*	48.21*	3.48*	1.85*
Vu *et al.*, 2012^39^	0, 1, 2, 3, 4, 8	6	156.31*	59.51*	4.33*	2.50*
summary			165.05	58.5	3.48	2.23

Timepoints in brackets were not sampled for all participants.

Source of data: ǂ, from paper; *, from individual-level data provided by authors; #, from graph-digitizing software.

At the 300 mg q12h and 600 mg q12h doses, PK sample collection was intensive across five studies and heterogeneity was lower (*I*^2^ <50% for *f*AUC_0–24_ and *fC*_min_ at both doses). However, data at these doses were reliant on a single centre (three out of five studies at both doses). Summary estimates for the 300 mg q24h and 600 mg q24h doses relied on sparse sampling from only two studies and results demonstrated a high degree of inter-study heterogeneity (*I*^2^ = 89%–97% for *f*AUC_0–24_ and 67%–99% for *fC*_min_).

### Monte Carlo simulation of the attainment of PK/PD targets

**Figure 4.**
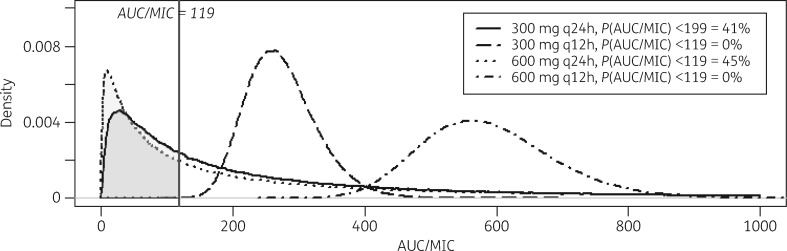
Probability density distributions of the attainment of linezolid *f*AUC_0–24_:MIC >119 mg/L/h (vertical line) in a Monte Carlo simulation of 100 000 patients at different doses of linezolid, based on a published MIC distribution and summary AUC_0–24_ from a meta-analysis of published data.

**Figure 5.**
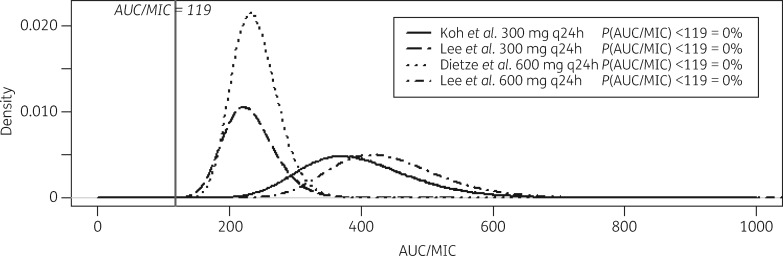
Probability density distributions of the attainment of linezolid *f*AUC_0–24_:MIC >119 mg/L/h (vertical line) in a Monte Carlo simulation of 100 000 patients at different doses of linezolid, based on a published MIC distribution and summary AUC_0–24_ in a sensitivity analysis imputing individual studies at the 300 mg q24h and 600 mg q24h doses separately.

Using the summary estimates of *f*AUC_0–24_ from the meta-analysis and the WT MIC distribution we assessed attainment of *f*AUC_0–24_:MIC >119 mg/L/h for each dose in a simulated population of 100 000 individuals (Figure 4).^30^ The efficacy target was attained in all simulated individuals at the 300 mg q12h and 600 mg q12h doses. The target was not attained for 41.0% and 44.6% of simulated individuals at the 300 mg q24h and 600 mg q24h doses, respectively. Given the high heterogeneity between studies at the 300 mg q24h and 600 mg q24h doses, we performed a sensitivity analysis by imputing each study at these doses into the simulation independently. In this analysis, the efficacy target was attained by all individuals in both studies at both doses (Figure 5).

Using the summary estimates for *fC*_min_ from the meta-analysis we simulated the attainment of *fC*_min_ <1.38 mg/L for each dose (Table 2). More than 98% of individuals at 600 mg q12h and at least 20% of individuals at all doses failed to achieve this target. Again, because of heterogeneity between studies at the 300 mg q24h and 600 mg q24h doses, we performed a sensitivity analysis, imputing the individual studies at these doses into the Monte Carlo simulations. Differences between attainment of the safety target when imputing studies individually were substantial (64.19% for Koh and Shim^34^ versus 94.95% for Lee *et al.*^11^ at 300 mg q24h and 97.87% for Dietze *et al.*^21^ versus 33.68% for Lee *et al.*^11^ at 600 mg q24h).

**Table 2.** Percentage of 100 000 simulated patients below a safety threshold, *fC*_min_ <1.38 mg/L, based on summary PK data for different linezolid doses

Dose	Percentage below 1.38 mg/L
300 mg q24h	75.47%
300 mg q12h	79.30%
600 mg q24h	72.53%
600 mg q12h	1.42%

## Discussion

Linezolid is an important drug in the management of RR-TB and MDR-TB, but its use is often limited by toxicity, prompting consideration of reduced dosing strategies. Our analysis is the first, to our knowledge, to provide summary PK data and simulate PK/PD target attainment to inform dose selection in clinical practice and clinical trials. We meta-analysed published data to generate summary estimates of plasma *f*AUC_0–24_:MIC and *fC*_min_ at different doses of linezolid, then performed Monte Carlo simulations based on these summary estimates to quantify attainment of putative PK/PD targets for efficacy and safety.

Current PK data on linezolid in TB patients are limited. Eight clinical studies, using four dosing strategies, were available for our analysis. These used variable, sometimes sparse, sampling schedules resulting in considerable heterogeneity between studies when meta-analysing data at 300 mg q24h and 600 mg q24h doses. Consequently, summary estimates for *f*AUC_0–24_ and *fC*_min_ at these doses are accompanied by wide standard deviations. Sensitivity analyses based on separate simulations for each study at these doses show that attainment of efficacy and safety targets is strongly influenced by inter-study heterogeneity. Consequentially, existing data do not definitively support any one dosing strategy and further prospective linezolid PK studies, ideally using standardized sampling schedules, are required. Nonetheless, important observations can be made from our analysis.

A linezolid dose of 1200 mg/day has recently been used alongside bedaquiline and pretomanid as part of the Nix-TB trial regimen (NCT02333799) on the basis of continued dose–response in an early bactericidal activity study. Preliminary results suggest that this regimen achieves good clinical outcomes, but 71% of patients have at least one dose interruption owing to toxicity.^40^ Prior PK data are unavailable for 1200 mg q24h, so we meta-analysed data for 600 mg q12h. In our simulations, 100% attainment of the efficacy target, but <1% attainment of the safety target, is consistent with the emerging Nix-TB results of high efficacy, but problematic, side effects. The ZeNix trial (NCT03086486) will test the efficacy and toxicity of 600 mg q24h versus 1200 mg q24h of linezolid within this regimen.

In search of a less toxic dosing regimen, prior meta-analyses support the clinical efficacy of linezolid 600 mg/day or lower.^9^^,^^10^ One lower-dose linezolid strategy is 300 mg q12h, for which our simulations described 100% efficacy target attainment and failure to meet the safety target in only 20.7% of patients. These results support preferential use of this dose. However, as many patients were from a single centre, generalizability of this finding will depend on prospective studies in other populations. Alternatively, once-daily dosing at 600 mg q24h is often advocated because of greater convenience. Our simulations were based on a meta-analysis of two studies and described only 55.5% efficacy target attainment and failure to meet the safety target in 27.5% of simulated patients. Assuming a half-life of 5 h, accumulation ratios of 1.03 and 1.23 are expected for q24h and q12h linezolid dosing regimens, respectively, so the AUC_0–24_ for linezolid may be up to 20% higher for 300 mg q12h than 600 mg q24h and this may have contributed to higher efficacy target attainment with the 300 mg q12h dose. However, as our sensitivity analyses show that heterogeneity of study results strongly influenced attainment of efficacy and safety targets in simulations at 600 mg q24h, further studies are required before judgement can be passed on this dosing strategy.

A lower linezolid dose of 300 mg q24h is used clinically, particularly in patients who have already reported side effects. We found limited PK assessment of this strategy. In simulations based on meta-analysis of data from two studies, efficacy target attainment and failure to meet the safety target were similar to 600 mg q24h at 59.0% and 24.5%, respectively. This demonstrates that effective therapy is possible at 300 mg q24h for some individuals, but that linezolid will cause some toxicity irrespective of dose alteration. As with 600 mg q24h, the high degree of heterogeneity in study results at this dose complicates these analyses and underlines the need for prospectively gathered PK data at this clinically important dose.

Overall, these data suggest that future clinical trials containing linezolid should evaluate multiple dosing regimens and that trials of alternative oxazolidinones that retain efficacy with lower toxicity are urgently needed. For instance, sutezolid has demonstrated greater antimycobacterial activity than linezolid in a whole-blood culture model, treatment shortening in a mouse model and sustained EBA_0–14_ in humans (which have not been demonstrated with linezolid), whilst demonstrating a more favourable PK/PD profile in terms of likely mitochondrial inhibition and apparently lower rates of toxicity in small, limited-duration, human studies.^8^^,^^41^^,^^42^ Trials of cyclical linezolid courses to maximize efficacy and then allow cumulative toxicity to abate should be considered; we could not assess this strategy in our analysis. Intermittent dosing strategies have been proposed, whereby a higher linezolid dose (e.g. 1200 mg) is given on alternate days to ensure efficacy target attainment, but allow longer periods of safety target attainment.^43^ Our data provide supportive evidence that the summary estimate of AUC_0–24_ for 600 mg q12h approximates a doubling of the 300 mg q12h and 600 mg q24h summary estimates for AUC_0–24_, but existing data do not allow us to comment on any improvements in safety target attainment with intermittent dosing. Whilst revised dosing strategies are being established, therapeutic drug monitoring may have a role in maximizing attainment of efficacy and safety targets for individual patients. Moreover, population PK models indicate that renal clearance accounts for up to 70% of inter-individual variation in linezolid levels, suggesting a potential benefit from initial dosing based on renal function, formulae for which have been proposed.^13^^,^^44^

In addition to highlighting the need for more PK data, this study has several limitations. Our putative PK/PD efficacy and safety targets may not be precise. The efficacy target was based on HFIM data in the absence of any measurement validated against clinical outcomes. The safety target was derived from one clinical study from Asia, with thrombocytopenia as the principal outcome.^28^ This may not be representative of overall linezolid toxicity. More robust linezolid PK/PD targets for TB therapy require prospective clinical evaluation. Secondly, the WT linezolid MIC distribution used for *f*AUC:MIC simulations was from drug-susceptible TB because there are no published linezolid MIC distributions for RR-TB or MDR-TB. However, MIC_50_ and MIC_90_ values for these populations are in broad agreement with the WT data.^31–33^ Additionally, the MIC testing for this distribution was conducted using Middlebrook 7H10 media and may not be representative of the distribution obtained using alternative media.^30^ Thirdly, development of linezolid resistance during therapy is an important outcome and may be a particular risk at lower doses.^45^ We have not yet simulated the attainment of resistance prevention PK/PD targets and future studies should seek to do this.

In conclusion, despite increased use of linezolid in RR-TB and MDR-TB treatment, there remains no consensus on optimal safe dosing. Current PK/PD data are insufficient to confidently provide a solution. Compared with the standard dose of 600 mg q12h, a dose of 300 mg q12h may retain efficacy with lower toxicity. Prospective clinical studies are required to test this proposition and to better assess once-daily dosing strategies.
